# Toronto Emergency Medical Services and SARS

**DOI:** 10.3201/eid1009.040170

**Published:** 2004-09

**Authors:** Alexis Silverman, Andrew Simor, Mona R. Loutfy

**Affiliations:** * Toronto Emergency Medical Services, Toronto, Ontario, Canada;; † Sunnybook and Women's College Health Sciences Centre, Toronto, Ontario, Canada;; ‡ McGill University, Montreal, Quebec, Canada;; § North York General Hospital, Toronto, Ontario, Canada

**Keywords:** letter, severe acute respiratory syndrome, outbreak, emergency medical services, management

**To the Editor:** The first appearance of severe acute respiratory syndrome (SARS) in China in November 2002 led to a worldwide epidemic by March 2003. On February 21, 2003, an index case of SARS, which led to 224 cases and 38 deaths, was diagnosed in Toronto. On March 14, four cases of atypical pneumonia in Toronto were epidemiologically linked to the SARS outbreak in China. On March 26, the Ontario Ministry of Health declared a provincewide medical state of emergency, which was lifted on May 17 when the SARS outbreak was thought to be over. However, 7 days later, several more cases of SARS were discovered in four Toronto hospitals, which caused a resurgence of the intensive precautionary measures throughout the healthcare sector. When the state of emergency was lifted on July 2, 2003, a total of 224 people in Toronto had been officially diagnosed with SARS, and 38 had died.

The SARS outbreak strained Toronto Emergency Medical Services (EMS), which worked 40 stations evenly divided among the city's four quadrants. Annually, Toronto EMS transports >140,000 patients to 17 acute-care hospitals, which makes it the largest and busiest municipal EMS in Canada. During the outbreak, Toronto EMS's 850 paramedics had 1,166 potential SARS exposures; 436 were placed in a 10-day home quarantine, which meant being isolated from those persons within the home, continuously wearing an N95 respirator, and taking their temperature twice a day. SARS-like illnesses developed in 62 paramedics, and suspected or probable SARS requiring hospitalization developed in 4 others. On March 26, almost all of the frontline staff of the city's northeast quadrant were sent home because of possible SARS exposure at a Toronto hospital (*1*). On May 22, when the outbreak's second phase began, >200 paramedics had contact with patients with SARS and were quarantined. These events seriously affected EMS and their staff.

Even before the SARS emergency was declared in Ontario, Toronto EMS was aware of a serious respiratory disease in the community. Because of an increase in "atypical pneumonia" cases, an advisory had been sent to all paramedics warning them to wear respirators, gowns, gloves, and goggles with all respiratory patients. The advisory was recalled in favor of the Provincial Directive; the Provincial Directive was also changed when SARS reemerged in May. While properly fitting and supplying 850 paramedics with respirators took several months, no paramedics became ill with SARS after these requirements were initiated, even without fit-testing all the respirators.

Although cleaning the emergency vehicles was a potential concern, the only important change was substituting the usual disinfectant of 3% hydrogen peroxide with virucidal effect in 10 minutes to a disinfectant of 7% activated hydrogen peroxide with virucidal effect in 5 minutes. Otherwise, normal procedures were followed and emergency vehicles were cleaned on their regular rotational basis.

During the outbreak, the EMS Healthcare Divisional Operations Centre became the emergency operations center for Toronto EMS. It had been designed to coordinate Toronto's operational response with other municipal and provincial health services. During this time, the province also created its own emergency operations center, to which representatives from both health services reported.

Within days of the provincial emergency, Toronto EMS, in conjunction with Toronto police and fire services, created the medical support unit that operated as an internal public health department for all paramedics and was responsible for their direction, education, support, and screening. If needed, paramedics were placed under work or home quarantine or precautionary symptom surveillance on the basis of their exposure history, symptoms, and treatment in an emergency department or SARS clinic if needed. The medical support unit used protocols developed by a base hospital medical director who, together with EMS staff, reviewed each paramedic's chart daily to make appropriate follow-up decisions. The medical support unit was a vital component in protecting the paramedics' health and welfare.

To sustain the optimal functioning of Toronto EMS, its headquarters was closed to frontline staff for the duration of the outbreak. All personnel had to be screened for SARS-like symptoms before entering, and all paramedics had to check themselves for signs and symptoms of a SARS-like illness before reporting for duty. Anyone with SARS-like symptoms had to report to the medical support unit and stop working in an EMS capacity.

To control the spread of SARS, the provincial government placed all interfacility transfers under the control of Toronto EMS through the creation of the Provincial Transfer Authorization Centre on March 29. Since then, the Provincial Transfer Authorization Centre has been responsible for ensuring that all nonemergency transfers are medically cleared to prevent patients with contagious diseases from being taken to a facility that is unprepared to receive them. The Provincial Transfer Authorization Centre now processes >1,200 requests daily and was an important factor in containing SARS.

Several lessons were learned from the SARS outbreak. First, an emergency plan must be in place before an outbreak occurs. Second, the ability to communicate quickly and easily with provincial and municipal health authorities was needed to ensure that the most up-to-date information concerning the outbreak was available. The intergovernmental relationships necessary for such rapid communication should be established in advance. Third, accurate and timely communication with frontline staff members is the best way to minimize their fears. Finally, personal protective equipment procedures should be maintained until assurance that the exposure risk is negligible. The SARS outbreak is unlikely an isolated occurrence; therefore, sound advance planning on the basis of experience will increase the ability to protect both EMS staff and the public in the future.
